# A plasma 3-marker microRNA biosignature distinguishes spinal tuberculosis from other spinal destructive diseases and pulmonary tuberculosis

**DOI:** 10.3389/fcimb.2023.1125946

**Published:** 2023-02-28

**Authors:** Qiang Liang, Weidong Jin, Zhigang Huang, Huquan Yin, Shengchun Liu, Liehua Liu, Xiangwei Song, Zili Wang, Jun Fei

**Affiliations:** ^1^ Department of Spinal Surgery, Yantai Yuhuangding Hospital, Yantai, China; ^2^ Department of Spinal Surgery, General Hospital of Ningxia Medical University, Yinchuan, China; ^3^ Department of Orthopedics, The Third People’s Hospital of Shenzhen, Shenzhen, China; ^4^ Department of Biochemistry, Inteliex Biomedical Corp, Tampa, FL, United States; ^5^ Department of Orthopedics, The Tenth People’s Hospital of Shenyang, Shenyang, China; ^6^ Department of Spine Surgery, The Third Affiliated Hospital of Chongqing Medical University, Chongqing, China; ^7^ Department of Orthopaedics, First Affiliated Hospital of Xinxiang Medical College, Weihui, China; ^8^ Department of Spine Surgery, Xi’an International Medical Center Hospital Affiliated to Northwest University, Xi’an, Shaanxi, China; ^9^ Department of Orthopedics, Affiliated Hangzhou Chest Hospital, Zhejiang University School of Medicine, Hangzhou, Zhejiang, China

**Keywords:** microRNAs, spinal tuberculosis, diagnostic model, discrimination, biomarker

## Abstract

Accurate spinal tuberculosis (TB) diagnosis is of utmost importance for adequately treating and managing the disease. Given the need for additional diagnostic tools, this study aimed to investigate the utility of host serum miRNA biomarkers for diagnosing and distinguishing spinal tuberculosis (STB) from pulmonary tuberculosis (PTB) and other spinal diseases of different origins (SDD). For a case-controlled investigation, a total of 423 subjects were voluntarily recruited, with 157 cases of STB, 83 cases of SDD, 30 cases of active PTB, and 153 cases of healthy controls (CONT) in 4 clinical centers. To discover the STB-specific miRNA biosignature, a high-throughput miRNA profiling study was performed in the pilot study with 12 cases of STB and 8 cases of CONT using the Exiqon miRNA PCR array platform. A bioinformatics study identified that the 3-plasma miRNA combination (hsa-miR-506-3p, hsa-miR-543, hsa-miR-195-5p) might serve as a candidate biomarker for STB. The subsequent training study developed the diagnostic model using multivariate logistic regression in training data sets, including CONT(n=100) and STB (n=100). Youden’s J index determined the optimal classification threshold. Receiver Operating Characteristic (ROC) curve analysis showed that 3-plasma miRNA biomarker signatures have an area under the curve (AUC) = 0.87, sensitivity = 80.5%, and specificity = 80.0%. To explore the possible potential to distinguish spinal TB from PDB and other SDD, the diagnostic model with the same classification threshold was applied to the analysis of the independent validation data set, including CONT(n=45), STB(n=45), brucellosis spondylitis (BS, n=30), PTB (n=30), spinal tumor (ST, n=30) and pyogenic spondylitis (PS, n=23). The results showed diagnostic model based on three miRNA signatures could discriminate the STB from other SDD groups with sensitivity=80%, specificity=96%, Positive Predictive Value (PPV)=84%, Negative Predictive Value (NPV)=94%, the total accuracy rate of 92%. These results indicate that this 3-plasma miRNA biomarker signature could effectively discriminate the STB from other spinal destructive diseases and pulmonary tuberculosis. The present study shows that the diagnostic model based on 3-plasma miRNA biomarker signature (hsa-miR-506-3p, hsa-miR-543, hsa-miR-195-5p) may be used for medical guidance to discriminate the STB from other spinal destructive disease and pulmonary tuberculosis.

## 1 Introduction

Tuberculosis is still a common disease in developing countries, with nearly 10 million new cases each year, of which about 2% involve the spine (Wang et al., 2016; Dunn and Ben Husien, 2018). Middle- and late-stage spinal tuberculosis often have severe kyphosis, which affects the appearance and may cause paralysis due to spinal cord compression. However, if spinal tuberculosis can be diagnosed in its early stage and given regular and sufficient anti-tuberculosis medication in time, the progress of this disease can be blocked. Doctors’ diagnosis of spinal tuberculosis is still mainly achieved by imaging examination and empirical cognition of clinical manifestations. Since spinal tuberculosis is very similar to other spinal infectious diseases in clinical and imaging manifestations, it is challenging to accurately diagnose spinal tuberculosis [3,4]. The primary deficiencies in the “gold standard” bacterial culture test in laboratory tests for diagnosing infectious diseases are: first, bacterial culture takes too long; second, the positive rate is too low. The modified Roche culture method is a clinical standard for culturing tuberculosis bacteria. It generally takes 2-5 weeks to produce the culture results, and the positive culture rate is only about 30% (Nussbaum et al., 1995; Garg and Somvanshi, 2011). Researchers have been looking for better methods to rapidly and accurately diagnose infectious diseases.

In recent years, the research on diagnostic biomarkers has made significant progress, and the research on miRNA has achieved gratifying results. miRNA is an endogenously produced small RNA in eukaryotic cells about 20-24 nucleotides in length, which specifically binds to 3’UTRs of its target mRNA to inhibit or completely block the gene expression of its target mRNA, plays a vital role in regulating cell physiology and pathogenesis. miRNA has high tissue specificity, spatiotemporal specificity, and stability and is very suitable as a diagnostic marker. In recent years, many scholars have tried to find diagnostic features in the blood for various diseases and found that miRNAs can be used as specific diagnostic markers for multiple conditions, such as tumors, immune disorders, and infectious diseases (Zhang et al., 2013; Correia et al., 2017). We infer that differentially expressed specific miRNAs also occur during the pathogenesis of spinal tuberculosis.

The present study discovered STB-specific candidate multi-biomarker combinations by Exiqon miRNA PCR Array in plasma samples of spinal tuberculosis patients and standard healthy control. Then, STB specific diagnostic model containing a multi-marker panel was developed by multivariate logistic regression method in the training sample set. After then, the diagnostic model based on the 3-plasma miRNA biomarker signature was validated using cohorts from spinal tuberculosis, other spinal destructive diseases, and pulmonary tuberculosis.

## 2 Materials and methods

### 2.1 Study design and population

A total of 423 samples were collected in 4 different clinics; who were diagnosed with spinal tuberculosis, active pulmonary tuberculosis, brucellosis spondylitis, malignant spine tumors, and pyogenic spondylitis by three senior doctors combining clinical manifestations, imaging, laboratory examinations, and histopathological examinations, and ordinary persons in the medical examination centers of hospitals mentioned above during the same period. Random sampling divided the samples into 3 phases (discovery, training, and validation). This research protocol was approved by the Ethics Committee of the General Hospital of Ningxia Medical University and implemented by the principles of the Declaration of Helsinki. All participants have signed informed consent. The research objects were patients hospitalized in the General Hospital of Ningxia Medical University, the Integrated Chinese and Western Medicine Hospital of Zhejiang Province, the Third People’s Hospital of Shenzhen, the Tenth People’s Hospital of Shenyang, the First Affiliated Hospital of Xinxiang Medical College, the Fourth People’s Hospital of Yinchuan from September 2016 to December 2018. There were no statistical differences in age and gender between the spinal tuberculosis group and the standard control group at various stages. The study population chart is shown in **Table 1**.

**Table 1:** Study population chart.

	CONT	BS	PTB	ST	PS	STB
**Discovery**	8	0	0	0	0	12
**Training**	100	0	0	0	0	100
**Validation**	45	30	30	30	23	45
**Total**	153	30	30	30	23	157

CONT, Healthy control; BS, Brucellosis spondylitis; PTB, Pulmonary tuberculosis; spinal tumor: ST, PS, Pyogenic spondylitis; STB, Spinal tuberculosis.

### 2.2 Sample collection

10 mL of blood was drawn from the cubital vein of all subjects in the morning of the next day after admission and placed in two EDTA tubes, respectively. The venous blood was centrifuged at 2500 rpm for 5 minutes after standing at 4°C for 30 minutes in the refrigerator. The clear supernatant (i.e., plasma) was gently extracted using a pipette and then put into 2 ml enzyme-free cryopreservation tubes. The miRNAs in plasma were separated immediately, or the tubes were placed in a refrigerator at -80°C as soon as possible.

### 2.3 Plasma miRNA isolation

Total RNA was isolated from 200 μl of plasma using a miRcute miRNA extraction kit (TIANGEN, China) according to the manufacturer’s protocol. RNA quality and quantity were assessed using a nanodrop spectrophotometer (ND-1000, Nanodrop Technologies), and RNA Integrity was determined by gel electrophoresis.

### 2.4 miRNA labeling and array hybridization

After quality control, the miRCURY™ Hy3™/Hy5™ Power labeling kit (Exiqon, Vedbaek, Denmark) was used according to the manufacturer’s protocol. The Hy3™-labeled samples were hybridized on the miRCURYTM LNA Array (v.19.0) (Exiqon) according to the array manual. And the data were collected using the Axon GenePix 4000B microarray scanner (Axon Instruments, Foster City, CA).

### 2.5 Array data processing and enrichment analysis of differentially expressed miRNAs

Scanned images from Axon GenePix, 4000B microarray scanner, were imported into GenePix Pro 6.0 software (Axon) for grid alignment and data extraction. Replicated miRNAs were averaged, and miRNAs with intensities>=30 in all samples were chosen to calculate the normalization factor. Expressed data were corrected with the background signal and then normalized using the quantile normalization (Chekka et al., 2022). After normalization, significant differentially expressed miRNAs between two groups were identified through Benjamin Hochberg (BH) corrected p-value (q value) was to be q< 0.1, and the absolute value of log2 fold change (FC) was set to be > 1.5. Finally, hierarchical clustering was performed to show distinguishable miRNA expression profiling among samples. To select the biologically function-related biomarker candidates, the functional enrichment analysis of significant differentially expressed miRNAs was performed using TAM 2.0 database (Li et al., 2018).

### 2.6 qRT-PCR analysis

The cDNA was synthesized using the miRcute Plus miRNA First-Strand cDNA Kit (Tiangen) according to the manufacturer’s protocol, and RT-qPCR was carried out using Roche 480 fluorescence quantitative PCR instrument (Roche) with cel-miR-39 as an external control (Hauser et al., 2012; Syring et al., 2015; Liu et al., 2019).

### 2.7 Statistical analysis

All statistical analyses were performed using R software 4.2.2 (R Foundation for Statistical Computing, Vienna, Austria) and GraphPad Prism 9.0 (San Diego, CA, U.S.A.). RT-qPCR results were expressed by Log^-delta Ct^ (delta Ct = Ct value of target miRNA-Ct value of cel-miR-39). Data distribution normality tests were performed by D’Agostino-Pearson algorism. The categorical variable data groups were compared using the chi-square test and Kruskal-Wallis test or t-test to compare the continuous data groups. The Chi-square test was used to detect the correlation between the relevant clinical data and each miRNA change. The multivariate logistic regression method established the diagnostic model to calculate the predictive value, and Youden’s J index determined the optimal classification threshold. Bilateral P<0.05 was considered significant.

## 3 Results

### 3.1 miRNA expression profile in the discovery cohort

STB-specific miRNAs in plasma samples were identified using the Exiqon miRNA PCR array platform in the discovery cohort, including STB patients (n=12) and the CONT group(n=8). Compared with the normal control group, 13 miRNAs were most significantly up-regulated or down-regulated in the spinal tuberculosis group with the following selection criteria: Benjamin Hochberg (BH) corrected p-value (q value)< 0.1 and the absolute value of log2 fold change (FC) were set to be > 1.5. There are three types of up-regulation: hsa-miR-199b-5p, hsa-miR-506-3p and has-miR-4726-3p; ten types of down-regulation: hsa-miR-195-5p, hsa-miR-190a-5p, hsa-miR-500a-3p, hsa-miR-27a-3p, hsa-miR-543, hsa-let-7b-5p, hsa-miR-3186-5p, hsa-miR-3188, hsa-miR-3192-5p and hsa-miR-3934-5p (**Table 2**). In the view of biological sense, these candidate miRNAs performed the disease functional enrichment analysis with the criteria of FDR<0.05. Functional categorization of these significantly changed miRNAs, whose expression might be regarded as STB-specific, demonstrates that nine miRNAs (>69%, hsa-miR-199b-5p, hsa-miR-506-3p, hsa-miR-195-5p, hsa-miR-190a-5p, hsa-miR-500a-3p, hsa-miR-27a-3p, hsa-miR-543, hsa-let-7b-5p, hsa-miR-3188z) are involved in various carcinoma disease signaling pathways. We selected the three most significantly changed miRNAs used as candidate biomarkers among these eight miRNAs. These miRNAs are hsa-miR-506-3p, hsa-miR-543, and hsa-miR-195-5p.

**Table 2:** Significantly changed 13 miRNA biomarker candidates.

miRNAs	P value	q value	Mean of CONT	Mean of STB	FC of log
hsa-miR-506-3p	0.004893	0.079605	6.434	8.084	1.65
hsa-miR-199b-5p	0.000023	0.02218	5.248	6.82	1.572
hsa-miR-4726-3p	0.000305	0.038061	5.415	6.92	1.505
hsa-miR-3934-5p	0.000876	0.041104	4.994	3.468	-1.525
hsa-miR-3186-5p	0.008091	0.097757	5.876	4.301	-1.575
hsa-miR-500a-3p	0.005488	0.083582	6.281	4.677	-1.605
hsa-miR-3192-5p	0.000061	0.02218	3.89	2.281	-1.608
hsa-let-7b-5p	0.002016	0.054522	12.33	10.51	-1.826
hsa-miR-27a-3p	0.004968	0.079816	4.681	2.681	-2
hsa-miR-190a-5p	0.0049	0.079605	4.434	2.262	-2.172
hsa-miR-3188	0.000312	0.038061	2.531	0.3069	-2.224
hsa-miR-195-5p	0.001424	0.047305	4.719	2.454	-2.265
hsa-miR-543	0.006239	0.088258	3.254	0.828	-2.426

FC, fold change; q value: adjusted p-values optimized by Benjamin Hochberg (BH) FDR method.

### 3.2 Diagnostic performance of selected candidate STB-specific miRNAs in the training cohort

**Figure 1:**
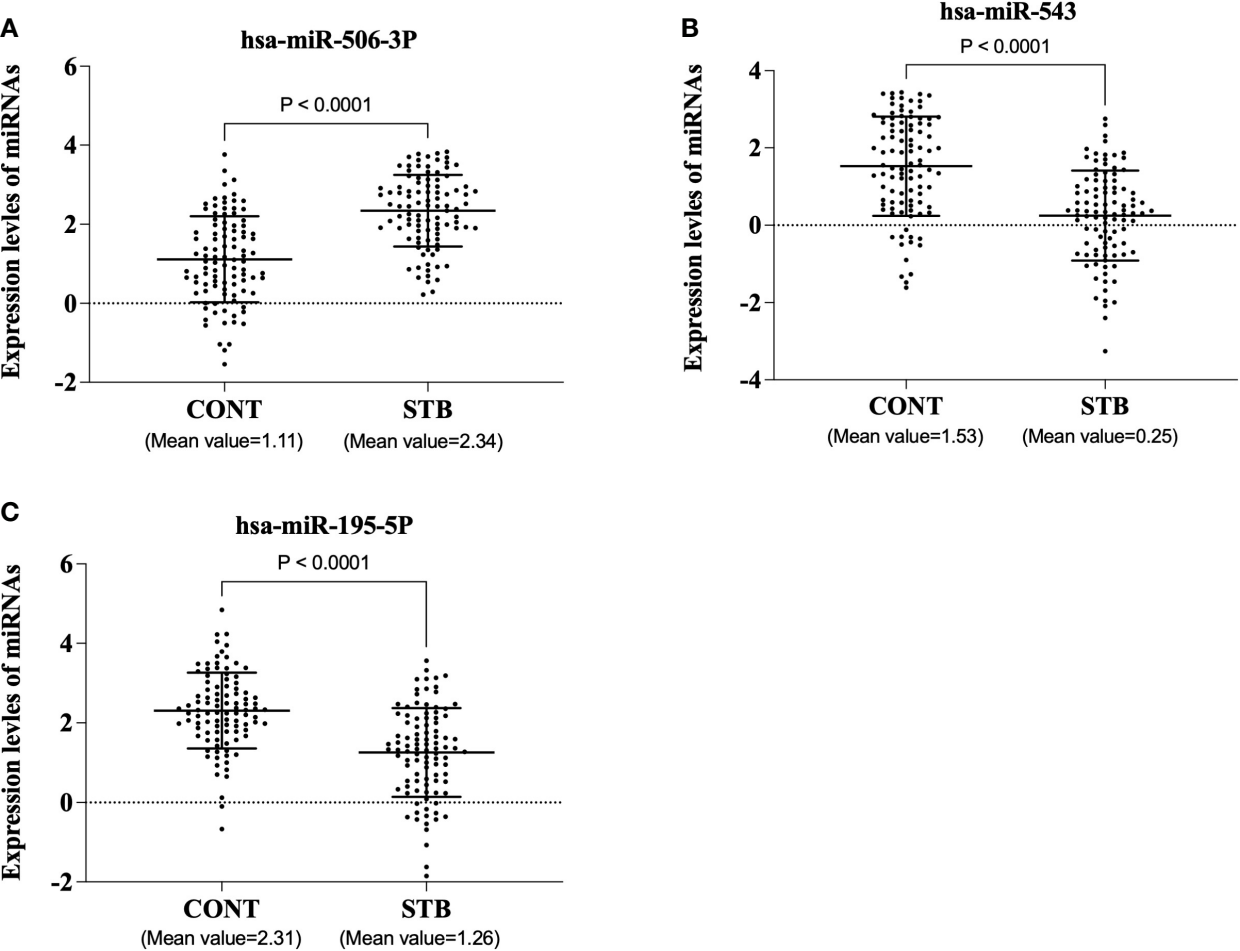
The expression levels of three miRNAs in the training cohort. The plasma samples were voluntarily collected, and miRNAs were isolated using a commercially available kit. qRT-PCR was performed according to the manufacturer’s protocol. The results were expressed as Log^-delta Ct^ (delta Ct = Ct value of target miRNA-Ct value of cel-miR-39). **(A)**. hsa-miR-506-3p **(B)**. hsa-miR-543 **(C)**. hsa-miR-195-5p.

**Figure 2:**
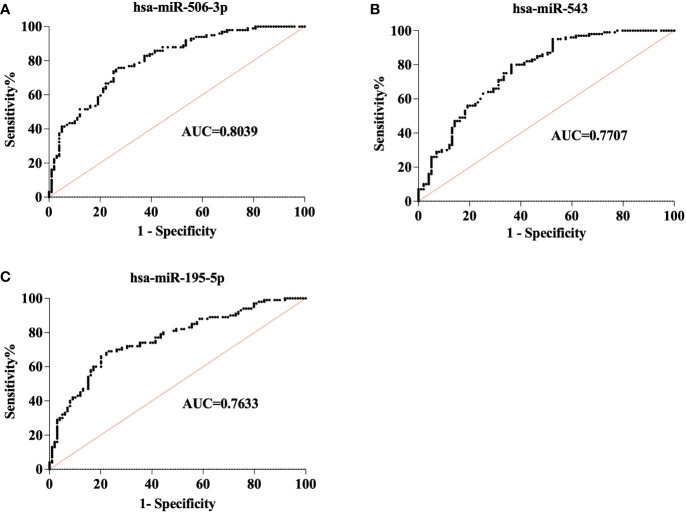
Receiver operating characteristic (ROC) analysis of 3 miRNAs in the training cohort. ROC curve shows the diagnostic performance of 3miRNAs in the training cohort (CONT = 100, STB= 100). **(A)**. hsa-miR-506-3p (AUC = 0.8039) **(B)**. hsa-miR-543(AUC=0.7707) **(C)**. hsa-miR-195-5p(AUC=0.7633).


Two hundred plasma samples (CONT, n=100; STB, n=100) in the training cohort were used to analyze the diagnostic performance of selected biomarker signatures. The qRT-PCR study showed that the expression of hsa-miR-506-3p, hsa-miR-543, and hsa-miR-195-5p were significantly (P<0.0001) changed compared to CONT (**Figure 1**). ROC curve analysis of individual miRNAs shows hsa-miR-506-3p has AUC=0.8039, hsa-miR-543 has AUC=0.7707, and hsa-miR-195-5p has AUC=0.7633, (**Figure 2**). For the next step, the diagnostic model for STB was developed using multivariate logistic regression based on the expression levels of 3 selected miRNAs as variables. The established multivariate logistic regression model is as follows:

$$\begin{array}{l} \log\left(\frac{p\left(STB\right)}{1-p\left(STB\right)}\right)=0.5863+1.055\left(\text{hsa}-\text{miR}-506-3\text{p}\right)\\ -0.8074\left(\text{hsa}-\text{miR}-543\;\right)-0.9694(\text{hsa}-\text{miR}-195-5\text{p}) \end{array}$$

**Figure 3:**
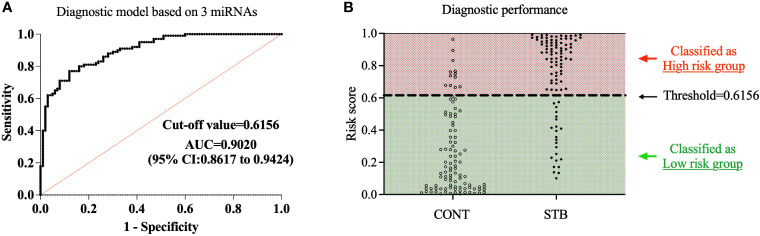
Development of diagnostic model based on a three miRNA signature. A diagnostic model for STB was developed using multivariate logistic regression based on the expression levels of 3 selected miRNAs as variables. **(A)**. The diagnostic accuracy of the diagnostic model was assessed by ROC analysis. **(B)**. The samples were divided into high-risk and low-risk groups based on the cutoff value of the diagnostic model; the population of CONT and STB groups was then classified into high-risk and low-risk groups.

For simplicity, we define the p(STB) value as the risk score. To interpret and evaluate a biomarker, the optimal threshold was determined by Youden’s J index field (Reiser, 2000; Wasserman, 2004). When the cut-off value is set as 0.6156, the maximum J value was archived with Negative predictive value (NPP)= 78.38% and Positive predictive value (PPP)=86.36%. Then we classified all the samples into high-risk and low-risk groups based on the cutoff threshold. ROC curve analysis shows that the current diagnostic model that contains three miRNA biosignatures demonstrated high accuracy of diagnosis of STB with AUC=0.9020, Sensitivity=87.9%, Specificity=76.0% (**Figures 3A, B**). The results indicate that the combination of three miRNA signatures has superior diagnostic performance than individual miRNAs.

### 3.3 Discrimination ability of the diagnostic model in validation cohort: Distinguish the STB from other spinal destructive diseases and pulmonary tuberculosis

**Figure 4:**
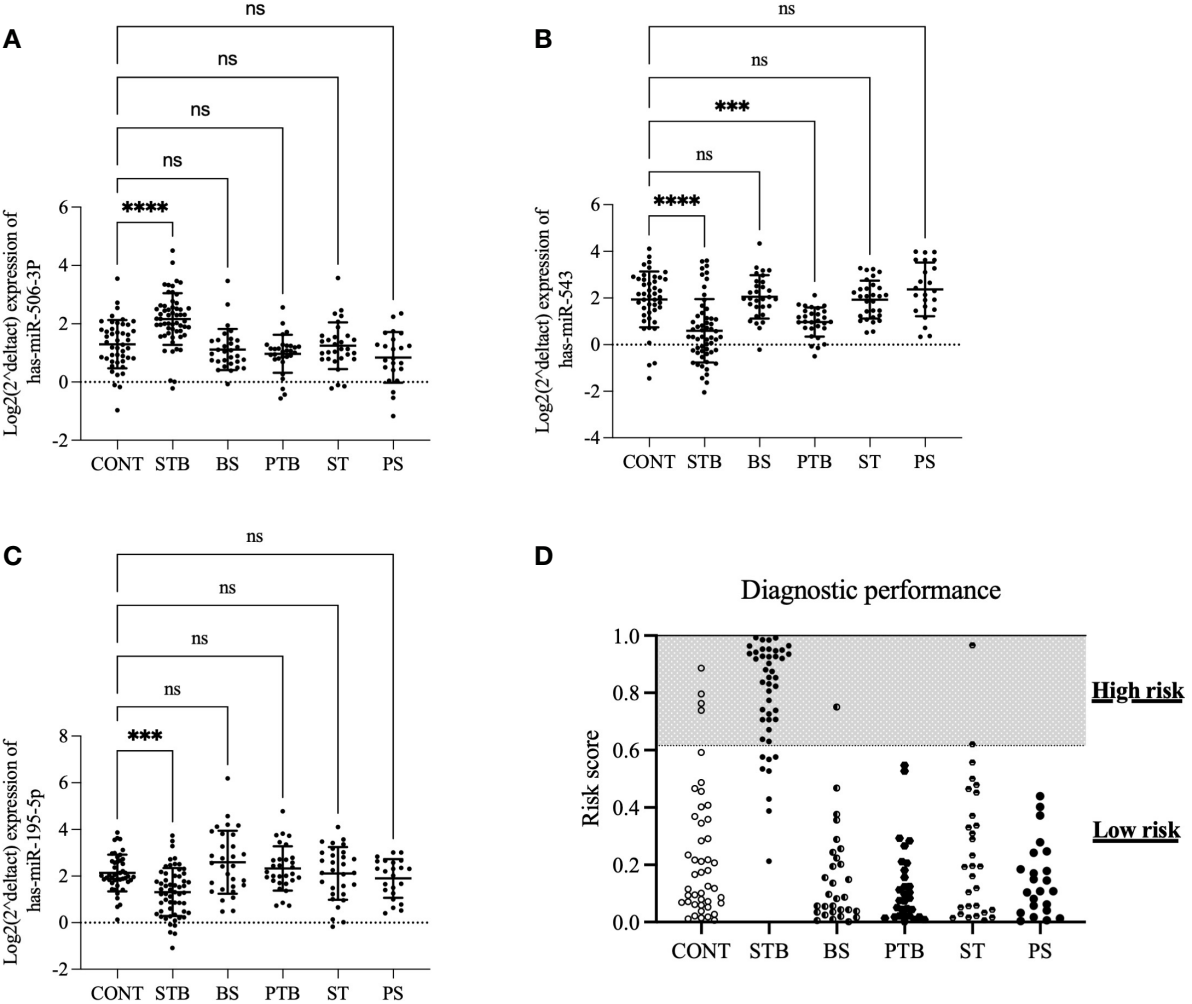
The expression profiles of 3 miRNAs and performance of the diagnostic model in the validation cohort. The expression levels of 3 miRNAs were assessed in the independent validation cohort, including CONT(n=45), STB(n=45), brucellosis spondylitis (BS, n=30), PTB (n=30), spinal tumor (ST, n=30) and pyogenic spondylitis (PS, n=23). **(A)**. hsa-miR-506-3p **(B)**. hsa-miR-543 **(C)**. hsa-miR-195-5p. A p-value was determined by the Kruskal-Wallis test **(D)**. the population of indicated groups was classified into high-risk and low-risk groups. ***P ≤ 0.001; ****P ≤ 0.0001; ns: P > 0.05.

Our next question is whether this diagnostic model based on a three-miRNA signature can distinguish spinal TB from PTB and other SDD. The expression levels of 3 miRNAs were assessed in the independent validation cohort, including CONT(n=45), STB(n=45), brucellosis spondylitis (BS, n=30), PTB (n=30), spinal tumor (ST, n=30) and pyogenic spondylitis (PS, n=23). As shown in **Figure 4**, the expression levels of hsa-miR-506-3p and hsa-miR-195-5p in STB are significantly different compared to CONT. However, there is no significant difference between CONT and other groups (**Figures 4A, C**). The expression level of hsa-miR-543 in STB is significantly different compared to CONT. There is no significant difference between CONT and other groups, except with the PTB group (**Figure 4B**). This means the expression changes of hsa-miR-506-3p, hsa-miR-543, and hsa-miR-195-5p are the STB specific and considering the diagnostic model is the combination of 3 miRNAs, these results indicate the diagnostic model based on three miRNA signatures may have a possibility to distinguish spinal TB from PDB and other SDD.

The same diagnostic model and optimal cut-off from the training cohort were applied to the analysis of diagnostic performance in these validation cohort groups to explore this possibility. The results showed that the current diagnostic model based on a three-miRNA signature could discriminate the STB from other SDD groups (**Figure 4D**), with sensitivity=80%, specificity=96%,

Positive Predictive Value (PPV)=84%, Negative Predictive Value (NPV)=94%, and the total accuracy is 92%.

### 3.4 Correlation analysis of the expression levels of 3 miRNAs with STB patient characteristics

The correlation of the three miRNAs with clinical indexes of STB patients, including age, sex, osteosclerosis, abscess formation, sequestration, disc destruction, C-Reactive Protein (CRP), and Erythrocyte Sedimentation Rate (ESR), was evaluated by chi-square test with Fisher’s exact test. Based on the average expression levels of 3 miRNAs, 157 patients with spinal tuberculosis were divided into a high-expression group (n=78) and a low-expression group (n=79). The results showed that the expression of hsa-miR-506-3p was significantly correlated with the formation of a paravertebral abscess (P=0.002); the expression of hsa-miR-543 was significantly associated with the rim osteosclerosis and sequestration (P=0.000; P=0.008); the expression of hsa-miR-195-5p was significantly correlated with the increase of CRP (P=0.002) (**Table 3**).

**Table 3:** Association of the expression levels of 3miRNAs with STB patient clinical characteristics.

Characteristics	hsa-miR-506-3p			hsa-miR-543			hsa-miR-195-5p		
	High	Low	P-value	High	Low	P-value	High	Low	P-value
Age(years)									
≤60	56	61	0.436	54	63	0.131	57	60	0.171
>60	22	18		24	16		21	19	
Sex									
Male	37	39	0.809	35	42	0.299	38	39	0.935
Female	41	40		43	37		40	40	
Osteosclerosis									
Yes	23	22	0.82	13	23	0	21	24	0.632
No	55	57		65	46		57	55	
Abscess formation									
Yes	62	45	0.002	56	51	0.33	52	56	0.568
No	16	34		22	28		26	23	
Sequestration									
Yes	24	22	0.688	19	35	0.008	22	24	0.765
No	54	57		59	44		56	55	
Disc destruction									
Yes	42	46	0.58	39	49	0.129	44	44	0.928
No	36	33		39	30		34	35	
CRP									
Normal	11	13	0.682	12	12	0.973	14	10	0.357
Elevated	67	66		66	67		64	69	
ESR									
Normal	21	17	0.296	18	20	0.743	27	11	0.002
Elevated	57	68		60	59		51	68	

## 4 Discussion

In the early 1980s, Chalfie discovered for the first time in nematodes that Lin-4 could play an essential role in the growth and development of larvae and the physiological process of egg production (Chalfie et al., 1981). Since then, miRNA research has gradually become a new hot spot. Studies have proved that miRNA has the following characteristics: first, miRNA has the feature of small molecular weight, so it is easy to diffuse from the lesion into plasma and other body fluids; second, because of its small number of bases, it is not easy to decompose so, it has better biological stability; third, miRNA mainly binds to the 3’untranslated region of the target mRNA entirely or partially, and respectively plays the role of degrading the target mRNA or inhibiting its transcription so, it has high tissue specificity and spatiotemporal specificity. These characteristics determine that miRNA is very suitable as a disease diagnostic marker.

With the advancement of high-throughput detection technology in recent years, scholars have studied miRNA expression in patients with various infectious diseases and achieved many gratifying results in field Fields (Verma et al., 2016; Baldassarre et al., 2017). Yi et al. found 92 differentially expressed miRNAs in the sera of patients with tuberculosis, of which 59 were up-regulated and 23 were down-regulated. The expression of miRNA-146 was significantly increased compared with the healthy group. Alipoor et al. (2019) found that the expression levels of miR-484, miR-425, and miR-96 in the serum exosomes of tuberculosis patients were significantly increased. Wiwanitkit et al (Wiwanitkit and Wiwanitkit, 2012). found that miRNA-29 could be used as a diagnostic marker for pulmonary tuberculosis. However, there has yet to be any previous study regarding the discovery and validation of plasma-circulating miRNAs in the STB patient. We convince that spinal tuberculosis is different from pulmonary tuberculosis in its onset site. During the pathogenesis, miRNAs involved in the pathophysiological processes of bone infection, bone formation, and bone destruction will indeed infiltrate into the blood in the blood vessel, causing changes in the expression of circulating miRNAs in plasma.

In the present study, we discovered the candidate miRNAs in the plasma samples of STB patients and normal healthy groups using a high-throughput array platform. The candidate miRNAs were trained and validated in a large sample cohort by qRT-PCR to develop the diagnostic model of spinal tuberculosis.

At the discovery stage, the Exiqon miRNA PCR array was used for high-throughput screening free miRNAs in the plasma of 12 patients with spinal tuberculosis and eight normal healthy controls. The results showed that 13 miRNAs were most significantly up-regulated or down-regulated in the spinal tuberculosis group (q<0.1, the absolute value of log2 fold change (FC) > 1.5). Disease functional enrichment analysis showed that eight miRNAs were enriched in biological function. We selected the three most significantly changed miRNAs used as candidate biomarkers among these eight miRNAs. Using multivariate logistic regression, we developed an STB-specific diagnostic model based on three selected biomarkers in the following training cohort. The performance analysis shows that the diagnostic model that contains three miRNA biosignatures demonstrated high accuracy of diagnosis of STB with AUC=0.9020, Sensitivity=87.9%, and Specificity=76.0%. In the independent validation cohort study, we found the established diagnostic model could effectively discriminate spinal tuberculosis from other spinal destructive diseases and pulmonary tuberculosis with sensitivity=80%, specificity=96%,

Positive Predictive Value (PPV)=84%, Negative Predictive Value (NPV)=94%, and the total accuracy is 92%. These results indicate that the established diagnostic model met the requirements as a diagnostic marker.

The previous study has shown that miR-543 can target and regulate Dickkopf 1 and Smad7, the essential proteins of the Wnt signaling pathway (Chen et al., 2018; Shen et al., 2019). Pan et al. found that the Wnt signaling pathway can regulate bone homeostasis by mediating YAP (Yes-associated protein) (Pan et al., 2018). Studies also show that miR-195-5p can directly control the expression of YAP (Yang et al., 2018). In the current study, we found that the low expression of miR-543 in patients with spinal tuberculosis was correlated with the sequestration and focal hyperplasia and sclerosis. It is possible that miR-543 can regulate the expression of Dickkopf 1, Smad7, and other proteins in spinal tuberculosis lesions, mediate YAP, and affect bone formation. Xia et al. (2020) found that miR-506-3p could inactivate the SIRT1/AKT/FOXO3a signaling pathway, FoxOs could indirectly promote the osteoclast activity and function by regulating the osteoblast OPG expression and movement; SIRT1 and P300 combined in the bone-derived pre-osteoclasts, which may lead to the deacetylation of NF-κB, inhibit the transcriptional activity of NF-κB, and inhibit the formation of osteoclasts (Huang et al., 2012). So, we speculate that miR-506-3p is essential in bone destruction and regeneration in spinal tuberculosis.

In the current multicenter study, we successfully discovered and verified a diagnostic model containing three miRNAs that could be used as bio-diagnostic markers for spinal tuberculosis, which can effectively discriminate spinal tuberculosis from other spinal destructive diseases and pulmonary tuberculosis. And one of the limitations of this study is that the patients included in this study are from different regions of China and can represent the basic situation of the Chinese. Still, all patients are of Asian descent, and it failed to include the samples of white, brown, and black people (Li et al., 2014; Maxwell et al., 2015; Telonis and Rigoutsos, 2018). However, when miRNAs are used as a diagnostic marker for certain diseases, there will be differences in diagnostic efficiency among different races (Weber et al., 2010; Zhang et al., 2013). Therefore, the diagnostic model needs to be further verified and optimized in a larger independent cohort to be used in the clinic. Although the sample size used in this study is sufficient to build the diagnostic model, future studies with larger sample sizes should better optimize the diagnostic efficiency of this model.

## Data availability statement

The array data presented in the study are deposited in the ArrayExpress Gene Expression Omnibus (GEO) repository, accession number is GSE225679.

## Ethics statement

The studies involving human participants were reviewed and approved by Ethics Committee of the General Hospital of Ningxia Medical University. The patients/participants provided their written informed consent to participate in this study.

## Author contributions

QL: Conceptualization, Methodology; ZH: Funding acquisition, Conceptualization, Methodology; WJ: Conceptualization, Methodology; HY: Data curation, Writing- Original draft preparation. SL: Investigation Methodology and Data curation; LL: Investigation Methodology and Data curation; XS: Investigation Methodology and Data curation; ZW: Funding acquisition, Supervision, Project administration, Writing- Reviewing and Editing; JF: Funding acquisition, Supervision, Project administration, Writing- Reviewing and Editing. All authors contributed to the article andapproved the submitted version.
